# A Rare Presentation of Levetiracetam-Induced Torsades De Pointes

**DOI:** 10.7759/cureus.40866

**Published:** 2023-06-23

**Authors:** Henry Mann, Josef Kusayev, Sagar Pandey, Binit Aryal, Isaac Solaimanzadeh

**Affiliations:** 1 Internal Medicine, One Brooklyn Health System Interfaith Medical Center, Brooklyn, USA; 2 Medicine, New York Medical College, Valhalla, USA

**Keywords:** ventricular fibrillation, hypomagnesemia, hypokalemia, cardiac arrest, qt interval prolongation, s: levetiracetam, drug-induced torsades de pointes, torsades de pointes (tdp)

## Abstract

Torsades de pointes occurs in the presence of a prolonged QTc interval, which has many congenital and acquired causes. Levetiracetam is a widely used anti-epileptic medication secondary to its favorable safety profile. We present a rare case of a 59-year-old male who developed torsades de pointes and cardiac arrest after levetiracetam administration. To our knowledge, there is only one other case report documenting torsades de pointes after levetiracetam administration, and our case report will be the first documenting cardiac arrest after levetiracetam administration.

## Introduction

Torsades de pointes, or "twisting of points", is a form of polymorphic ventricular tachycardia that occurs in the setting of a prolonged QTc interval. The incidence of torsades is approximately .16% per year, and its mortality rate is approximately 10-20%. Its etiology can be grouped into congenital long QT syndromes and into acquired causes of prolonged QT interval-like medications, electrolyte disturbances, cardiac conditions, and endocrine disorders [1]. Levetiracetam is an anti-epileptic medication for patients with seizures and for seizure prophylaxis post craniotomy/traumatic brain injury and has been widely used since its introduction in 1999 secondary to its favorable safety profile as compared with other anti-epileptic medications [2]. We describe a unique case of a 59-year-old man who, after receiving levetiracetam, experienced hypokalemia/hypomagnesemia, a prolonged QT interval, torsades de pointes, ventricular fibrillation, and cardiac arrest. To our knowledge, there have been only 12 case reports published to date documenting that levetiracetam prolonged QT intervals and only one other case report documenting torsades de pointes after levetiracetam administration; our case report will be the first to document cardiac arrest after levetiracetam administration.

## Case presentation

**Figure 1:**
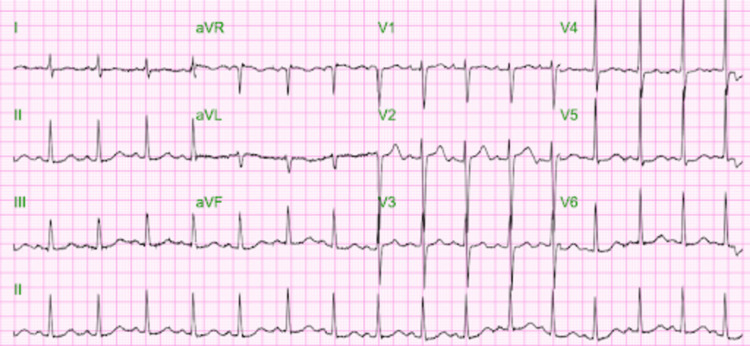
EKG on admission EKG reveals normal sinus rhythm with a QTc interval of 441 milliseconds

Our patient is a 59-year-old male with a past medical history of a seizure disorder who was brought to the emergency department from home for weakness. He had no known allergies or surgeries. Family history was non-contributory. Home medications included 250 milligrams of valproic acid orally every 12 hours, which the patient reported non-compliance with. Vitals were stable on admission, and physical examination was unremarkable. Remarkable labs on admission can be found in the table below (Table 1). Electrocardiogram (EKG) on admission (Figure 1) revealed a normal sinus rhythm and a corrected QT (QTc) interval of 441 milliseconds.

**Table 1:** Laboratory findings on admission µL - microliter, g - gram, dL- deciliter, fL - femtoliter, PLT - platelet, U - unit, L - liter, mg - milligram, mL - milliliter, min - minute, m2  - square meter, mmol - millimole, µIU - micro international unit, ng - nanogram

Laboratory test	Results	Normal range
White blood cells	4.5 cells/µL	4,500 - 11,000 cells/µL
Hemoglobin	9.1 g/dL	11.0 - 15.0 g/dL
Hematocrit	29.8%	35 - 46%
Mean corpuscular volume	89.2 fL	80 - 100 fL
Platelets	136 PLT/µL	130,000 - 400,000 PLT/µL
Aspartate aminotransferase	18 U/L	5 - 34 U/L
Alanine transaminase	19 U/L	10 - 55 U/L
Total bilirubin	0.3 mg/dL	0.2 - 1.2 mg/dL
Blood urea nitrogen	10.1 mg/dL	9.8 - 20.1 mg/dL
Creatinine	.73 mg/dL	0.57 - 1.3 mg/dL
Estimated glomerular filtration rate	99.5 mL/min/1.73m^2^	>=90.0 mL/min/1.73m2
Sodium	140 mmol/L	136 - 145 mmol/L
Potassium	3.7 mmol/L	3.5 - 5.1 mmol/L
Phosphorus	2.7 mg/dL	2.3 - 4.7 mg/dL
Magnesium	1.8 mg/dL	1.6 - 2.6 mg/dL
Thyroid-stimulating hormone	1.68 µIU/ml	0.465 - 4.680 µIU/ml
Free thyroxine	1.78 ng/dL	0.78 - 2.19 ng/dL

In the emergency room, the patient had an episode of a tonic-clonic seizure that lasted less than two minutes with a return to baseline, and about 20 minutes later, the patient developed a second episode of a tonic-clonic seizure that also lasted less than two minutes. In total, the patient received 1000 milligrams of levetiracetam intravenously and 4 milligrams of lorazepam intramuscularly to control his seizures. EKG performed after these seizures and levetiracetam/lorazepam administration revealed a QTc interval of 646 milliseconds; the patient was placed on a telemetry monitor, and repeat labs were immediately drawn, which showed a potassium level of 2.9 millimoles per liter, and a magnesium level of 1.3 milligrams per deciliter. The patient was soon found to have torsades de pointes on the telemetry monitor and suddenly went into cardiac arrest with ventricular fibrillation. Return of spontaneous circulation and conversion to sinus rhythm was achieved immediately following defibrillation. He was intubated during his cardiac arrest for airway protection and was upgraded to the intensive care unit. In the intensive care unit, potassium and magnesium were supplemented, and the patient was started on 750 milligrams of levetiracetam intravenously daily. Despite supplementation, potassium and magnesium levels continued to remain low, and the QTc interval continued to remain prolonged. Levetiracetam was eventually switched to 500 milligrams of Divalproex daily, after which potassium/magnesium levels normalized, and the QTc interval was found to be 456 milliseconds. The patient was eventually extubated and managed with bilevel-positive airway pressure ventilation and downgraded to the telemetry unit. In the telemetry unit, his potassium/magnesium levels remained within normal limits, and his QTc interval continued to be approximately 450 milliseconds; he had no more episodes of seizures or torsades de pointes. He was eventually weaned off of bilevel-positive airway pressure ventilation and discharged on 500 milligrams of Divalproex daily to follow-up outpatient.

## Discussion

Torsades de pointes, or "twisting of points", is a form of polymorphic ventricular tachycardia characterized by rapid, irregular QRS complexes "twisting" around the EKG baseline that occurs in the setting of a prolonged QT interval and can quickly lead to ventricular fibrillation and death. Its etiology can be grouped into congenital long QT syndromes and acquired causes of a prolonged QT interval, like medications, electrolyte disturbances, cardiac conditions, and endocrine disorders [1]. Repolarization in myocardial cells is primarily mediated by the efflux of potassium ions and the arrest in the influx of sodium and calcium ions. Conditions predisposing to prolonged QT intervals and subsequent torsades de pointes interfere with the current of these ions to prolong repolarization [3]. Congenital long QT syndrome can be caused by mutations in many genes encoding these cardiac ion channels [4]. Antiarrhythmics, antipsychotics, tricyclic antidepressants, and macrolide/fluoroquinolone antibiotics are some commonly implicated drugs for prolonging QT intervals [5]. Hypokalemia and hypomagnesemia are also both risk factors for the development of prolonged QT interval [6]. Hypomagnesemia causes QT interval prolongation due to sodium-potassium pump inhibition and subsequent change in membrane potential [7]. 

Levetiracetam has been widely used as an anti-epileptic since its introduction in 1999, secondary to its favorable safety profile as compared with other anti-epileptic medications [2]. However, there have been multiple case reports documenting levetiracetam-induced hypokalemia and hypomagnesemia [3,8-9]. Additionally, a literature review published in 2022 found twelve case reports and two studies that reported a prolonged QT interval caused by levetiracetam [10]. One case report reported torsades de pointes in a 24-year-old female with undiagnosed long QT syndrome who had her levetiracetam dose increased [11]. Other case reports also described improvements in potassium/magnesium levels and QT interval after levetiracetam discontinuation [9,12]. Our case report will be the first to our knowledge to describe levetiracetam-induced QT interval prolongation, torsades de pointes, and cardiac arrest in the background of hypokalemia and hypomagnesemia.

Administration of levetiracetam and the subsequent development of hypokalemia/hypomagnesemia, a prolonged QTc interval, torsades de pointes, ventricular fibrillation, and cardiac arrest helps to establish levetiracetam as the likely cause of these developments. Furthermore, potassium/magnesium levels normalized, and the QTc interval returned almost to normal once levetiracetam was discontinued in our patient. The absence of the history of administration of other potential QT interval-prolonging medications like antiarrhythmics, macrolides, fluoroquinolones, antipsychotics, and tricyclic antidepressants further narrows down the differential.

## Conclusions

Our case of levetiracetam-induced QTc interval prolongation and torsades de pointes leading to cardiac arrest is unique because, to our knowledge, there have been only twelve case reports published to date documenting that levetiracetam prolonged QT intervals and only one other case report documenting torsades de pointes after levetiracetam administration; our case report will be the first documenting cardiac arrest after levetiracetam administration. Levetiracetam-induced torsades de pointes, while not fatal in this case, is a serious adverse effect that can quickly lead to death. This case report highlights the importance of recognizing torsades de pointes as a rare but possible adverse effect of levetiracetam.
